# A Concept Analysis of Collaborative Practices in Mental Health

**DOI:** 10.1192/j.eurpsy.2026.10771

**Published:** 2026-07-31

**Authors:** C. Laranjeira, E. Pinheiro, A. K. Severo, E. Franco

**Affiliations:** 1 School of Health Sciences; 2 Centre for Innovative Care and Health Technology, Polytechnic University of Leiria, Leiria, Portugal; 3 Federal University of Rio Grande do Norte, Natal, Brazil

## Abstract

**Introduction:**

Collaboration in mental health care is crucial for executing a model focused on the psychosocial rehabilitation of individuals through multidimensional interventions that include various stakeholders and sectors of society to address their needs. Notwithstanding the advantages indicated by scientific data, numerous obstacles to collaborative care persist, and practitioners continue to grapple with adjusting their practices. The present circumstances necessitate organization and the formulation of sound action plans to address issues, which in turn requires a comprehensive understanding of collaborative practices in mental health care (CPMHC) and their conceptual limits.

**Objectives:**

A conceptual analysis study was conducted to establish a working definition of CPMHC.

**Methods:**

This study employed the Walker and Avant concept analysis methodology. This encompasses the identification of defining conceptual attributes, antecedents, consequences, and empirical referents. A literature search was conducted from November 2024 to February 2025 across three databases (Medline, CINAHL, and LILACS), focusing on papers published from 2010 to 2024.

**Results:**

Thirty 30 papers were examined. The essential attributes included effective communication, relationship development, shared responsibility for care, hierarchical adaptability, coordination among services, providers, and the community, oversight and assessment of care processes, and consideration of diverse sociocultural contexts.

**Conclusions:**

This concept analysis aids in directing future research and policy formulation about collaborative approaches in mental health, taking into account individual, relational, institutional, and social dimensions. More research is needed to enhance the comprehension of collaborative practices in mental health, considering the intricacies of social relations and systemic injustices.

**Disclosure of Interest:**

None Declared

